# TorsionAnalyzer: exploring conformational space

**DOI:** 10.1186/1758-2946-5-S1-P3

**Published:** 2013-03-22

**Authors:** Christin Schärfer, Tanja Schulz-Gasch, Matthias Rarey

**Affiliations:** 1 Center for Bioinformatics (ZBH), Bundesstr. 43, 20146 Hamburg, Germany; 2 F. Hoffmann-La Roche Ltd., 4070 Basel, Switzerland

## 

Molecular conformations are used in a wide range of virtual screening applications to represent the conformational flexibility of a molecule. As the underlying conformational model has a major influence on the results of these applications, a closer insight into the conformational space of molecules is very important.

We developed a new interactive graphical software tool for conformation analysis called TorsionAnalyzer. The tool uses a predefined set of over 450 SMARTS patterns to analyze the torsion angles of an input conformation. Each pattern describes a torsion angle and is associated with frequency histograms derived from CSD [1] and PDB [2] data. For each pattern a list of allowed torsion angles was automatically derived from the torsion histograms. Rotatable bonds of a conformation are colored according to their classification into usual, borderline and unusual torsion angles, using the list of allowed angles from the corresponding patterns. This allows the user to see at a glance if one of the torsion angles of an examined conformation is out of the ordinary. The TorsionAnalyzer supports easy modification of the existing SMARTS patterns as well as the preparation and storage of different sets of patterns for different sets of molecules.
